# Do functional walk tests reflect cardiorespiratory fitness in sub-acute stroke?

**DOI:** 10.1186/1743-0003-3-23

**Published:** 2006-09-29

**Authors:** Ada Tang, Kathryn M Sibley, Mark T Bayley, William E McIlroy, Dina Brooks

**Affiliations:** 1 Department of Physical Therapy, Faculty of Medicine, University of Toronto, Toronto, Canada; 2 Toronto Rehabilitation Institute, Toronto, Canada

## Abstract

**Background and purpose:**

The Six-Minute Walk Test (6MWT) has been employed as a measure of functional capacity, but its relationship to cardiorespiratory fitness in stroke is not well established. Gait speed measured over short distances is commonly used as an index of walking competency following stroke. We evaluated the relationship between the 6MWT, aerobic fitness (VO_2_peak) and walking competency in sub-acute stroke.

**Methods:**

Thirty-six individuals (mean age ± SD, 64.6 ± 14.4 years; time post-stroke 16.2 ± 13.3 days) were evaluated using the 6MWT (distance, speed, heart rate), a maximal exercise test (VO_2_peak, heart rate, exercise test duration), and walking competency using a five meter walk (speed, symmetry ratio). Correlation analyses were used to examine the relationships between these outcomes.

**Results:**

There was a strong correlation between the 6MWT and five meter walk velocity for preferred (r = 0.79) and fast (r = 0.82) speed (p < 0.001). On average, the 6MWT speed was faster than the preferred gait speed (94.9 cm/s vs. 83.8 cm/s, p = 0.003), but slower than the fast-paced walk (115.1 cm/s, p < 0.001). There was significant though more moderate association between 6MWT distance and VO_2_peak (r = 0.56, p < 0.001) and exercise test duration (r = 0.60, p < 0.001).

**Conclusion:**

The speed selected during the 6MWT was strongly related to the velocities selected during the five meter walk distance (intermediate to the selected preferred and fast speeds). Although the 6MWT may be challenging to the cardiorespiratory system, it appears to be more strongly influenced by potential limits to walking speed rather than cardiorespiratory capacity. As a result, this test is not, by itself, an adequate measure of aerobic fitness early after stroke.

## Background

Stroke is the leading cause of adult disability in North America [1,2]. Functional ambulation is often compromised [3-6], and reduced independence in walking is a commonly reported concern among stroke survivors [7]. This is further complicated by reductions in cardiorespiratory fitness after stroke [4,8-10], changes in neuromotor control [5] and increased energy demands associated with performing everyday activities [6,10]. These issues underscore the need for valid testing procedures to evaluate aerobic fitness and walking ability following stroke.

The measurement of maximal oxygen consumption during graded exercise testing is the gold standard for evaluating cardiorespiratory fitness [11]. However, stroke-related impairments such as changes in strength and sensory function may limit the use of graded maximal exercise tests with this population. Exercise testing is also expensive and time- and resource-intensive, and testing equipment is not readily available in most clinical settings. As an alternative, walk tests were designed as an objective measure of functional status and have been employed as a surrogate measure of cardiorespiratory fitness among several populations. In individuals with cardiorespiratory conditions for whom these tests were originally designed, correlations between Six-Minute Walk Test (6MWT) distance and VO_2_max range from 0.51 to 0.90 [12]. As there are multiple factors that may contribute to walk test performance among stroke survivors, the relationship between the 6MWT and aerobic capacity is not well established among stroke survivors, with associations ranging from none [9] to low (0.40, p < 0.005) [3] in chronic stroke and high correlation (0.84, p < 0.001) [4] in sub-acute stroke. In light of this variability, there is a need to better understand the determinants of the 6MWT that may influence its potential utility as a sub-maximal measure of cardiorespiratory fitness after stroke.

The relationship between the 6MWT and aerobic fitness in the stroke population may be confounded by limitations in walking competency arising from neuromotor control challenges. Another commonly used index of walking ability is gait speed measured over short distances (5–10 meters), which is considered primarily dependent on neuromotor control rather than aerobic fitness. Preferred (self-selected) and maximum gait speed is easily measured in the clinical setting, and preferred gait speed is commonly used as an outcome measure of walking competency in stroke rehabilitation, having been shown to be able to detect minimally clinically relevant change (responsive) in the sub-acute stroke population [13].

The purpose of this study was to examine the relationships between 6MWT, aerobic fitness (VO_2_peak) and walking competency (five meter walk) in sub-acute stroke (medically stable and < 3 months post). This work has important clinical implications for the evaluation of walking ability and cardiorespiratory fitness early post-stroke. As exercise training becomes increasingly recognized as an integral component of stroke rehabilitation programs during this important stage of recovery, valid and efficient tools for specifically evaluating aerobic and functional outcomes of interest are needed.

## Methods

The study procedures were followed in accordance to institutional guidelines and were approved by the local university and hospital research ethics committees. Informed written consent was obtained from all study participants. This study was part of a larger trial examining the effect of exercise in the sub-acute stroke population.

### Participants

Thirty-six patients with stroke were recruited from a rehabilitation facility and were included if they were able to provide informed consent, understand the evaluation procedures, walk at least five meters independently, have a Chedoke-McMaster Stroke Assessment (CMSA) [14] leg impairment score greater than 2 (where active voluntary movement is present without facilitation), and were less than three months post-stroke upon entry into the study. Participants were excluded if they exhibited any contraindications to maximal exercise testing as outlined by the American College of Sports Medicine (ACSM) [15], or musculoskeletal impairments or pain which would limit the ability to perform the tests.

Participant characteristics noted included demographic information, lesion type and location, time post-stroke, degree of neurologic deficit (motor, sensory, aphasia, apraxia, neglect) using the National Institutes of Health Stroke Scale [16], functional ability using the Functional Independence Measure [17], and level of leg impairment using the CMSA, where a score of 1 indicates flaccid paralysis, 3 describes a limb where spasticity and weakness are marked and 7, the maximum score, indicates normal limb function including complex movement patterns with appropriate muscle timing and coordination [14].

### Measures

All measures detailed below were performed during inpatient rehabilitation, with all but two participants closer to discharge. Typical duration of rehabilitation is 4–5 weeks, and includes physical, occupational, speech and language therapy 2–5 days per week.

### A) Six-minute walk test (6MWT)

Standardized instructions [18] were given to walk as far as possible over a 30-meter course in six minutes. Heart rate (HR) and rating of perceived exertion (modified 0–10 Borg scale) [19] were noted at the start and end of the test, as well as the use of walking aids. No encouragement was provided during the test. The distance covered in six minutes was the primary outcome of this test. Because of the known practice effect with the 6MWT in the cardiorespiratory population, participants were familiarized with the test with at least one practice trial before the actual measurement was taken. Gait speed, in cm/s, was determined [(distance covered during the 6MWT (m)/360 s) × 100].

### B) Cardiorespiratory fitness

A semi-recumbent cycle ergometer (Biodex Medical Systems, Shirley, NY) was used during the maximal exercise test and was selected to avoid the limitations in achieving aerobic capacity in sub-acute stroke using a walking or treadmill program. The ramp protocol included a two-minute warm up at 10 watts with a target cadence of 50 revolutions per minute, followed by progressive 5-watt increases in power output every minute. This protocol was designed specifically for this study, considering issues with strength and fatigability post-stroke and anticipating a total test time of 8–10 minutes. Participants were familiarized with the testing protocol with at least one practice trial prior to the actual test. A metabolic cart (AEI Technologies, Pittsburgh, PA) measured respiratory gas exchange with calculated averages at 30-second intervals. Blood pressure was monitored using an automated system (SunTech Medical, Morrisville, NC), as was heart rate with a heart rate monitor (Polar Electro Inc, Woodbury, NY). A 5-lead electrocardiogram (Remco Italia, Milano, Italy) was monitored for abnormalities. The foot on the participants' hemiparetic side was secured to the pedal if necessary. The test was terminated according to ACSM guidelines [15], or if the participant was unable to maintain the required pedaling rate despite encouragement. Peak oxygen uptake (VO_2_peak) and peak heart rate were determined as the highest values reached from the calculated 30-second averages from the exercise test.

### C) Walking competency (Five meter walk)

Spatiotemporal aspects of walking were measured using a pressure-sensitive mat, five meters in length (CIR Systems, Clifton, NJ). Thirty-four out of 36 participants were evaluated for walking competency due to initial problems in equipment availability. Three sets of walking trials were performed at preferred pace. A subset of 30 participants also performed three trials walking at their maximally comfortable speed (fast pace). The use of walking aids was noted. A research assistant provided safety supervision during each trial. The primary outcome measure was gait speed (cm/s), which was averaged across the three trials at each speed.

Gait symmetry ratio based on the preferred walk was computed [(paretic leg swing time/stance time)/(non-paretic leg swing time/stance time)]. A symmetry ratio between 0.9 and 1.1 (the 95% confidence interval for symmetry of gait in healthy adults) indicated a symmetrical gait pattern [20].

### Data analysis

Descriptive statistics were performed on all measures. Paired *t*-tests were used to determine significant differences between 6MWT speed and five meter walk gait speed (preferred and fast). Correlation analyses were performed to determine the relationships between 6MWT distance and VO_2_peak, 6MWT speed and five meter walk speeds (preferred and fast), and 6MWT distance and gait symmetry ratio. An unpaired *t*-test was performed to determine the difference between 6MWT distances between participants with respect to gait symmetry; one-way analysis of variance determined differences in 6MWT between levels of leg impairment. To determine predictors for 6MWT distance, stepwise multiple regression analysis was performed. Statistical significance was set at p < 0.05.

## Results

Participant characteristics are presented in Table 1. Participants were admitted to rehabilitation 16.2 ± 13.3 days post stroke, and were tested 50.3 ± 17.0 days post-stroke. Nineteen participants did not require any gait aids for walking, nine used a single point cane and eight used a two-wheeled walker or rollator. Results from the 6MWT, maximal exercise test and five meter walk are presented in Table 2. Due to issues with testing equipment for three individuals, gas analysis was not available for the maximal exercise test and as such, VO_2_peak was not measured. However, peak HR data was available for all 36 participants.

**Table 1:** Participant characteristics

		**Mean**	**SD**	**Range**
Male/Female, n	14/22			
Type of stroke, n				
Ischemic/Hemorrhagic/Unknown	25/10/1			
Hemiparetic side, n				
Right/Left/Bilateral	17/17/2			
Medications				
β-blockers/ACE inhibitors/None/Both	6/17/11/2			
Co-morbidities				
Hypertension	25			
Cholesteremia	12			
Diabetes mellitus	4			
Coronary artery disease	8			
Chronic obstructive pulmonary disease	3			
History of smoking	11			
Age, years		64.6	14.4	19–90
National Institutes of Health Stroke Scale*		2.8	2.3	0–10
Chedoke-McMaster Stroke Assessment Leg score		5.1	1.0	4–7
Functional Independence Measure		110.8	9.2	77–125

* Data missing for 6 participants

**Table 2:** Results from the 6MWT, maximal exercise test and five meter walk

	**n**	**Mean**	**SD**	**Range**
Six-Minute Walk Test				
Distance (m)	36	341.6	107.9	90–503
Speed (cm/s)	36	94.9	30.0	25.0–139.7
End heart rate (bpm)†	36	97.7	18.8	63–148
Maximal exercise test				
VO_2_peak (ml·kg^-1^·min^-1^)	33	12.3	3.1	6.6–19.2
Peak heart rate (bpm)†	36	115.0	25.9	70–175
Exercise test duration (min)	36	8.7	4.2	1.8–20.5
Gait speed (cm/s)				
Preferred	34	83.8	32.1	35.2–151.3
Fast	30	115.1	38.0	47.5–179.8
Gait symmetry ratio				
Preferred	34	1.11	0.24	0.63–1.73
Fast	30	1.13	0.20	0.82–1.71

† Beats per minute

### Relationship between 6MWT and cardiorespiratory fitness (VO_2_peak)

**Figure 1:**
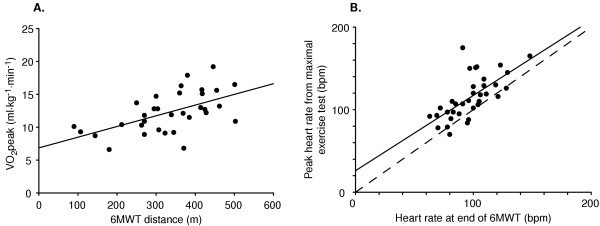
Relationship between A) 6MWT distance and VO_2_peak (r = 0.56, p < 0.001), and B) end heart rate from 6MWT and peak heart rate from maximal exercise test (r = 0.66, p < 0.001), in beats per minute (bpm). Dashed line represents line of identity.

Correlation analyses revealed 'moderate' associations between 6MWT distance with VO_2_peak (r = 0.56, p < 0.001) and between heart rate achieved at the end of 6MWT and the peak heart rate during the maximal exercise test (r = 0.66, p < 0.001) (Figure 1). Duration of the maximal exercise test was also moderately correlated to 6MWT distance (r = 0.60, p < 0.001). Average heart rate achieved at the end of the 6MWT was 85% of the peak heart rate from the maximal exercise test (97.7 ± 19.7 versus 115.1 ± 25.9 beats per minute) (t(35) = 5.37, p < 0.001) and 60% of age-predicted maximal heart rate (162 8 ± 1.7 beats per minute) (t(35) = -22.4. p < 0.001).

### Relationship between 6MWT and walking competency (gait speed, symmetry) and CMSA

**Figure 2:**
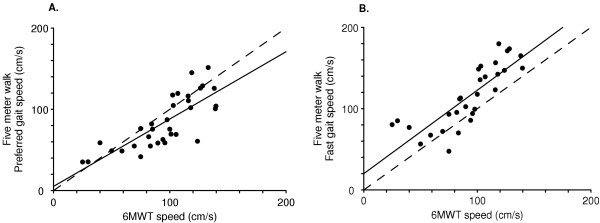
Relationship between 6MWT speed and A) preferred gait speed (r = 0.79, p < 0.001), and B) fast gait speed (r = 0.82, p < 0.001). Dashed line represents line of identity. Gait speed was faster during the 6MWT compared to preferred gait speed (t = -3.17, p = 0.003), but slower than the fast-pace walk (t = 5.6, p < 0.001).

The associations between gait speed from the 6MWT and preferred- and fast-paced walks were stronger (r = 0.79, p < 0.001 and r = 0.82, p < 0.001, respectively) (Figure 2). Gait speed during the 6MWT was faster than preferred gait speed from the five meter walk (94.9 ± 30.0 versus 83.8 ± 32.1 cm/s) (t(33) = 3.17, p = 0.003), but slower than fast-paced five meter walk (115.1 ± 38.0 cm/s) (t(29) = 5.62, p < 0.001).

**Figure 3:**
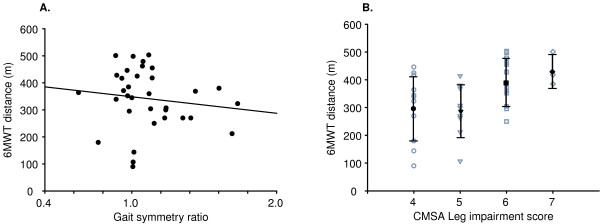
A) Relationship between gait symmetry ratio at preferred pace 6MWT distance (r = -0.26, p = 0.13), and B) CMSA individual scores (open symbols) and means and standard deviations (closed symbols and error bars) and 6MWT distance. There were no differences in 6MWT speed between participants with symmetrical versus asymmetrical gait patterns (t = 1.53, p = 0.14) and between those with different CMSA leg impairment scores (F = 2.17 p = 0.11).

Average symmetry ratios calculated based on the preferred- and fast-paced five meter walks are presented in Table 2. The association between 6MWT distance and preferred walk symmetry ratio was not statistically significant (r = -0.31, p = 0.24) and there was no significant difference in distance walked between symmetrical and asymmetrical groups (t(32) = 1.55, p = 0.13). There were wide ranges in 6MWT scores at all levels of CMSA leg impairment scores (Stage 4: 301.1 ± 114.2 m (90–446 m), Stage 5: 291.3 ± 94.1 m (107–414 m), Stage 6: 396.4 ± 86.0 m (250–503 m), Stage 7: 434.7 ± 59.8 m (385–501 m)). There was no significant difference in 6MWT distance between CMSA leg impairment scores (F (3,32) = 2.17 p = 0.11) (Figure 3).

### Determinants of 6MWT distance

Variables were entered into a stepwise multiple regression model to determine the predictors of 6MWT performance. Variables entered into the model included: measures from the maximal exercise test (VO_2_peak, peak heart rate and exercise test duration), preferred- and fast-paced five meter walk speeds, presence or absence of beta-blockade, and the number of medical conditions. The five meter fast walk (r = 0.81, p < 0.001) and exercise test duration (r = 0.87, p = 0.004) accounted for 65.4% and 9.7% of the variance in 6MWT distance. None of the remaining variables, when included in the model, led to a statistically significant improvement in the model prediction of 6MWT variance.

## Discussion

The relationships between gait speed, functional walk tests and aerobic capacity are not well established and have only received limited attention in chronic stroke. This study contributes to the current body of literature that reports associations between the 6MWT and measures of cardiorespiratory fitness and walking competency after stroke, and is the first study to make comparisons between short-distance walk and functional walk test gait speeds for individuals in the early post-stroke phase. There were strong relationships between speeds during the 6MWT and five meter walk, but the relationship between VO_2_peak and the 6MWT distance was modest, suggesting that aerobic fitness is a moderate contributor of distance walked on the 6MWT early after stroke.

Observed results from the 6MWT are comparable to other values in the stroke literature, slightly higher than some (316 m, 5.5 ± 4.9 y post-stroke [3] and 302 m, 29.2 ± 11.0 d post-stroke [4]) and lower than others (378 m, 3.5 ± 2.0 y post-stroke [9]), and provide further evidence that functional ambulation is compromised after stroke. Gait speed measured over short distances [4,9] is also similar to those previous reports. With respect to the correlation between 6MWT speed and preferred gait speed, Eng and colleagues reported a high correlation (0.92, p < 0.01) between self-selected gait speed and 6MWT distance for chronic stroke survivors, and interestingly, found that these individuals will pace themselves identically during a 6MWT and during the longer 12-Minute Walk Test [21]. Kelly et al. also reported similarly high correlations between 6MWT distance and self-selected and maximal gait velocities (r = 0.91 and r = 0.89, respectively) in individuals tested early after stroke [4]. Unlike the present study, comparisons of gait speed measured over short distances and 6MWT speed were not made in either of these previous studies. It has previously been reported that "comfortable" ten meter gait speed is faster than average 6MWT speed, suggesting that 6MWT performance may be dependent on aerobic capacity although somewhat independent of locomotor control, and that walking ability may be overestimated if gait speed is measured only over short distances [22]. In contrast, our findings show that preferred five meter gait speed is slower than 6MWT speed and would *underestimate *walk test performance, despite high correlations between the two. However, 6MWT speed was not as fast as the fast-paced five meter walk.

Low VO_2_peak values, approximately 60% of age-matched non-stroke populations [15], are comparable to findings from other studies [4,8] and confirm that aerobic fitness is compromised in the early stages post-stroke. The finding that HR values at the end of the 6MWT were 85% of those achieved during the maximal exercise test suggests that the walk test is challenging to the aerobic system; further, since 6MWT HR was only 60% of age-predicted maximal HR highlights the degree of de-conditioning that occurs soon after stroke. Arguably, pre-morbid fitness may have already been compromised prior to stroke onset due to the presence of cardiovascular co-morbidities and medication use or low activity levels, which may also account for the altered HR response. An earlier study focusing on sub-acute stroke reported a stronger correlation between 6MWT distance and VO_2_peak/age-predicted VO_2_max (r = 0.84, p < 0.001) [4], compared to the modest relationships between 6MWT distance with VO_2_peak (r = 0.56, p < 0.001) and exercise test duration (r = 0.60, p < 0.001) found in the current study. While compromised fitness may affect the distance walked on the 6MWT, stroke survivors likely have additional contributing impairments that limit both walking ability and performance on a maximal exercise test. Likewise, modest relationships are reported between 6MWT distance and measures of aerobic capacity in the cardiorespiratory domain [12]. In contrast, two studies investigating the relationship between the 6MWT and VO_2_peak with chronic stroke survivors found little [3] to no relationship [9], highlighting the range in exercise and functional performance within the stroke population. Walk tests were originally developed to evaluate the ability of individuals with cardiorespiratory conditions to maintain sub-maximal levels of intensity at durations intended to represent daily activities. Stroke-specific issues, however, such as balance and neuromotor control issues, may limit 6MWT performance more than cardiorespiratory fitness [3,21]. The present study contributes to the current knowledge by considering maximal exercise test results, gait speed, medication use and health status as potential determinants of 6MWT performance. Stepwise multiple regression analysis revealed that the five meter fast walk was the most important predictor of 6MWT distance accounting for 65% of the variance, while exercise test duration accounted for 10%. Consistent with the findings by Pang et al [3], VO_2_peak was not a significant determinant of 6MWT distance, confirming our hypothesis and providing further evidence that 6MWT distance does not adequately reflect cardiorespiratory fitness in the stroke population. Results from this multivariate analysis must be interpreted with caution however, due to the relatively small sample size.

Alternative functional walk tests with pre-determined test durations, such as the Long Distance Corridor Walk test, have been shown to achieve greater levels of cardiorespiratory effort among healthy elderly [23] and might be considered for application with individuals in the early post-stroke phase. Arguably, exercise testing on a cycle ergometer provides a potentially less confounded evaluation of cardiorespiratory fitness compared to walk tests, as the effects of some stroke-related impairments are minimized with the body weight supported and feet stabilized in the pedals. Future research could compare modality-specific differences (treadmill versus cycle ergometry) in evaluating aerobic capacity in the early post-stroke phase. The participants' ability to perceive effort levels due to altered sensation in the limbs may lead to different effort levels exerted during aerobic versus 6MWT assessments.

The current results highlight the large range of functional compensation in spite of significant physical impairment in the sub-acute stroke population. The wide range and lack of association between 6MWT distance and symmetry ratio suggests that gait symmetry is not a significant contributor to functional ambulation. Further, while there was no statistically significant difference between 6MWT distances across CMSA scores, there was an upward trend of longer distances with higher scores. However, there was a wide range of distances walked by individuals at a CMSA Stage 4 (90 to 446 m), with some participants in the range of individuals at a CMSA Stage 7, providing additional evidence of functional adaptation in spite of moderate levels of leg impairment and residual spasticity. A limitation of the current study is the relatively small sample size; with a larger sample, the association between 6MWT distance and symmetry ratio and CMSA scores may be significant.

Although the 6MWT was evaluated based on the "gold standard" maximal exercise test, there remains the unanswered question of the ideal method for evaluating fitness in sub-acute stroke. Performance on maximal exercise tests may depend on the severity of neurological impairment from stroke and subsequent reduction in functional muscle mass and oxidative capacity of the paretic muscles [10]. Additionally, conducting a maximal exercise test is expensive and resource-intensive and as such, these tests may be of limited use in the typical clinical setting. Sub-maximal measures of cardiorespiratory fitness may offer more cost- and time-effective and clinically feasible means of evaluation, but the validity and reliability of such tests have yet to be established in sub-acute stroke. Furthermore, these findings do not address a key question of whether these walking tests predict or correlate with other factors such as strength and power, neurological status, dynamical balance control, behavioral factors and the amount of community activity and independence in daily living activities. Certainly, tests based on evaluating aerobic demand using walking tasks need to be applied cautiously and may only be suitable for a subset of stroke patients. The findings in the current study suggest the need for both a maximal exercise test to measure cardiorespiratory fitness and the 6MWT to evaluate functional capacity in the physical assessment of individuals with stroke.

## Conclusion

The results from this study suggest that although the 6MWT may challenge the cardiorespiratory system in sub-acute stroke survivors, it is representative of the ability for functional ambulation and is not an adequate measure of aerobic fitness alone. The findings demonstrate positive correlations between gait speeds obtained from various measures of walking function and, more importantly, highlight differences between them. Early after stroke, individuals tend to walk at a faster, but not maximal, speed during functional walk tests compared to their comfortable pace. This suggests that gait velocity measured over short distances is not predictive of performance on the 6MWT. Findings from the current study have implications for the selection of assessments evaluating walking ability and aerobic capacity in sub-acute stroke, and for understanding the contributions of various factors, such as neuromotor control and cardiorespiratory fitness, on walking function.

## Competing interests

The author(s) declare that they have no competing interests.

## Authors' contributions

AT, as primary author, participated in study design, was responsible for writing the manuscript, data collection, analysis and interpretation. KMS participated in data collection and analysis and interpretation. MTB participated in study design, assisted with medical screening and data collection and interpretation. WEM conceived of the study, assisted with data analysis and interpretation. DB conceived of the study, assisted with data analysis and helped draft the manuscript. All authors have read and approved the final manuscript.
